# Primary Disseminated Pre-extensively Drug-Resistant Tuberculosis of the Lungs, Pleura, Chest Wall, and Abdomen: The World’s First Case

**DOI:** 10.7759/cureus.42281

**Published:** 2023-07-21

**Authors:** Sankalp Yadav

**Affiliations:** 1 Medicine, Shri Madan Lal Khurana Chest Clinic, Moti Nagar, New Delhi, IND

**Keywords:** abscess, mtb (mycobacterium tuberculosis), disseminated tuberculosis, pre-extensively drug-resistant tuberculosis, pre-xdr

## Abstract

Drug-resistant tuberculosis is a perpetual threat to public health. In recent years, there has been an increase in the number of cases of this deadly *Mycobacterium tuberculosis* infection. The present case is a very rare case of primary disseminated pre-extensively drug-resistant tuberculosis of the lungs, pleura, chest wall, and abdomen in a 19-year-old Indian female patient who presented with fever, cough, abdominal pain, and anterior chest wall swelling. The diagnosis was established by a detailed laboratory and radiological workup. An all-oral longer regimen was initiated per national guidelines and according to her weight.

## Introduction

Tuberculosis is an constant threat to public health [1]. This disease is prevalent in countries like India [2]. The incidence and prevalence stand at 188 and 312 per 0.1 million population, respectively [3]. There has been an increase in the number of cases recently, which could be attributed to the efforts of the National Tuberculosis Elimination Program aimed at notifying all cases, both from the public and private sectors [4].

Drug-resistant tuberculosis is a serious issue related to tuberculosis control [5]. A surge in the total number of drug-resistant tuberculosis cases has been noted in recent years [6]. As per the India TB report 2023, there are four categories of drug-resistant tuberculosis: multi-drug resistant/ rifampicin resistant-tuberculosis (MDR/RR-TB), pre-extensively drug-resistant tuberculosis (pre-XDRTB), isoniazid (INH)-resistant tuberculosis, and extensively drug-resistant tuberculosis (XDR-TB) [7].

Pre-extensively drug-resistant tuberculosis is tuberculosis in which resistance to rifampicin (MDR/RR-TB) and any fluoroquinolone is detected [7]. The data further shows that smear microscopy was done for about 13914910 presumptive tuberculosis patients during the year 2022, and about 4.5% of them i.e., 631,683 were diagnosed with tuberculosis [7]. Additionally, of the 55,004 second-line line-probe assays for the year 2022, there were 14,206 cases of fluoroquinolone resistance [7]. Also, second-line liquid culture-drug susceptibility testing conducted on 10,143 samples led to the detection of 2,411 cases of pre-extensively drug-resistant tuberculosis [7]. Nevertheless, 93% of these pre-extensively drug-resistant tuberculosis cases (11198/12002) were put on treatment regimens [7].

Herein, a case of a 19-year-old Indian female is described. She presented with complaints of fever, cough with expectoration, abdominal pain, and anterior chest wall swelling. A diagnosis of primary disseminated pre-extensively drug-resistant tuberculosis of the lungs, pleura, chest wall, and abdomen was made, and she was initiated on an all-oral longer regimen as per the national guidelines.

## Case presentation

In 2020, a 19-year-old Indian female of low socioeconomic status presented with complaints of fever, cough with expectoration, abdominal pain, and anterior chest wall swelling. She was asymptomatic 20 days prior when she had a low-grade intermittent fever without chills or rigors. She also had a cough for 15 days, which was associated with thick, yellow-colored, non-blood-tinged expectoration. She complained of abdominal pain for 10 days. The pain was insidious in onset, around the umbilicus, intermittent, and not associated with any aggravating or relieving factors. Additionally, she had developed a painful swelling on the right anterior chest wall over the sternum one week prior to presentation.

There was no history of trauma, seizures, or remarkable weight loss. She was a student, and there was no history of tuberculosis in her or in any of her contacts. There was no history of migration, substance abuse, stays at refugee camps, or night shelters. Moreover, there was no history of medical or surgical intervention.

The findings of a general examination of the patient included a temperature of 98.6°F, a pulse of 78 per minute, a blood pressure of 110/70 mm of Hg, a respiratory rate of 19 breaths per minute, a weight of 69 kg, and an oxygen saturation (SpO2) of 99% in room air. Local examination revealed a 4 X 5 cm cystic swelling over the manubrium sterni slightly on the right side. This swelling was smooth in consistency, tender to the touch, slightly mobile, irreducible, non-fluctuant, and positive on transillumination. There was no clubbing, icterus, cyanosis, koilonychia, pallor, or lymphadenopathy. Her systemic examination was remarkable for a dull percussion note over the right lung with decreased breath sounds and decreased tactile and vocal fremitus on auscultation. The abdominal examination was remarkable for tenderness in the umbilical region. The rest of the systemic examination was within normal limits.

Along with other routine blood tests, she underwent sputum fluorescent microscopy (Rhodamine auramine staining for *Mycobacterium tuberculosis*), a chest radiograph, a cartridge-based nucleic acid amplification test (CBNAAT) of the sputum, and an ultrasound of the chest and the whole abdomen.

**Figure 1:**
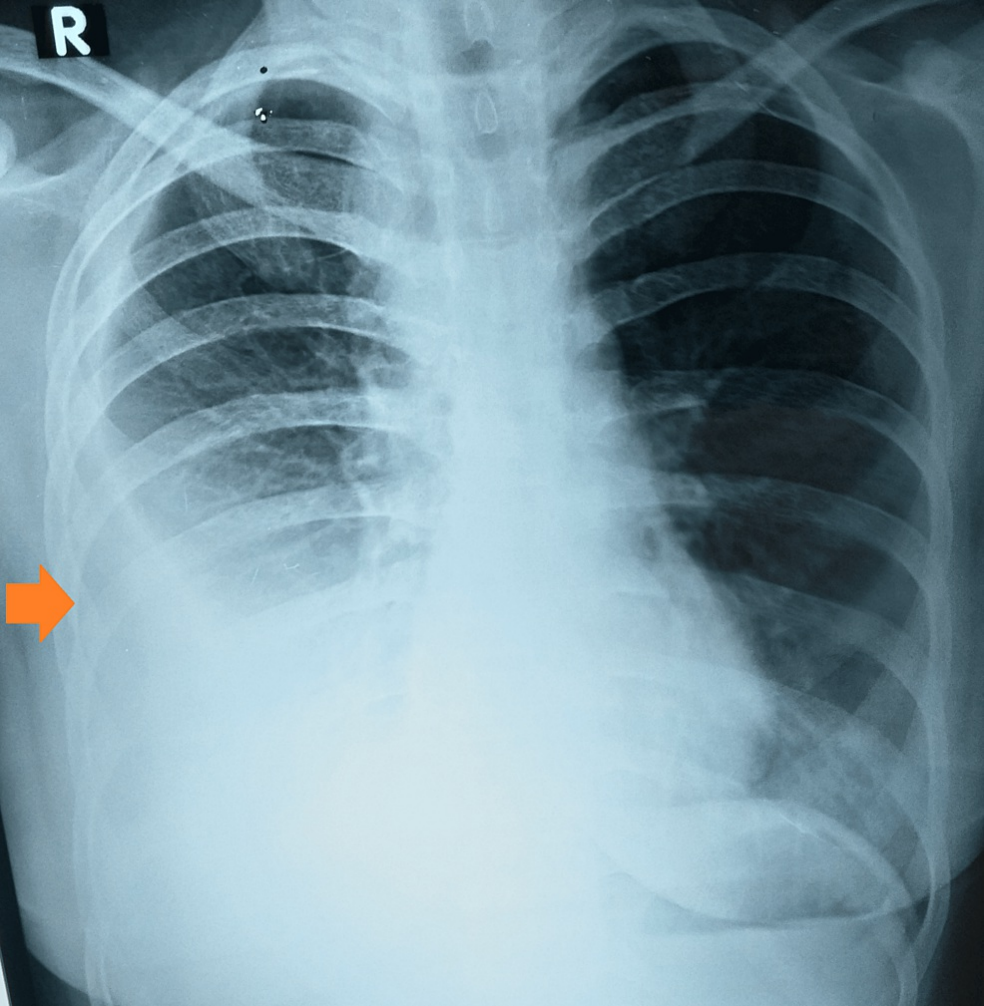
Chest radiograph (P-A) view suggestive of right-sided pleural effusion P-A: postero-anterior

The results were conclusive for tuberculosis, with sputum fluorescent microscopy positive (1+) for *Mycobacterium tuberculosis* and a low detection of *Mycobacterium tuberculosis *on CBNAAT with resistance to rifampicin. An additional sputum sample was sent for the line probe assay and culture for drug susceptibility testing. Her chest radiograph was suggestive of right-sided pleural effusion (Figure 1).

**Figure 2:**
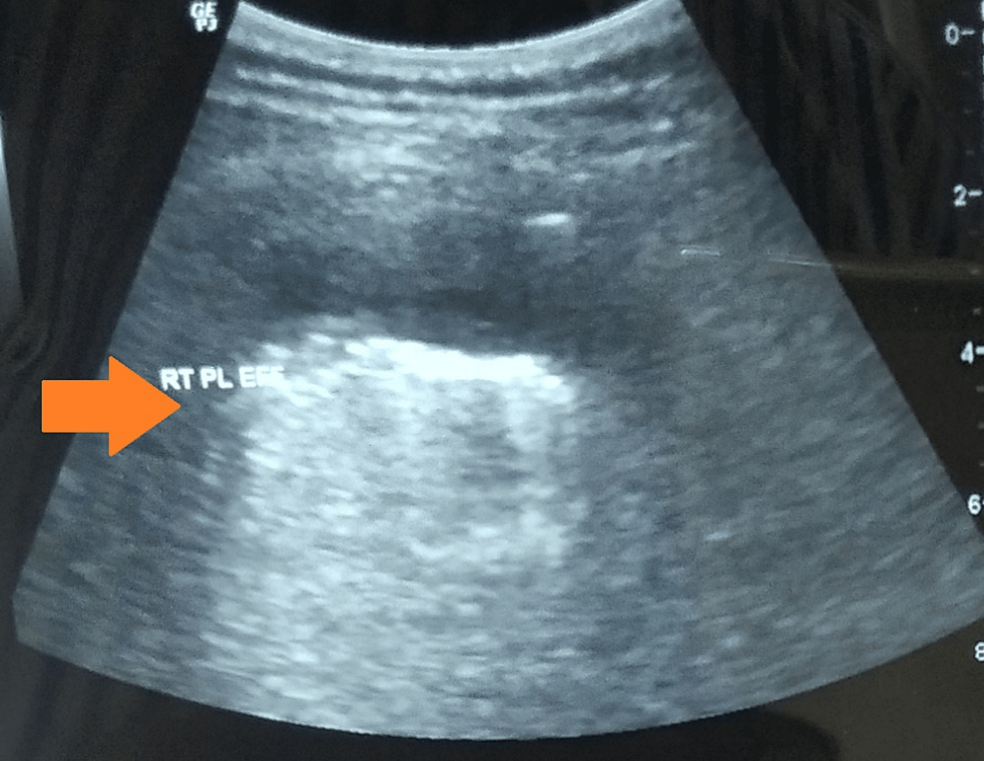
USG-chest revealing a 70.8 X 59.1 X 12.5 mm pleural effusion (arrow) USG: ultrasonogram

An ultrasound of the chest revealed a 70.8 X 59.1 X 12.5 mm sized pleural effusion (about 27-28 cc) (Figure 2).

**Figure 3:**
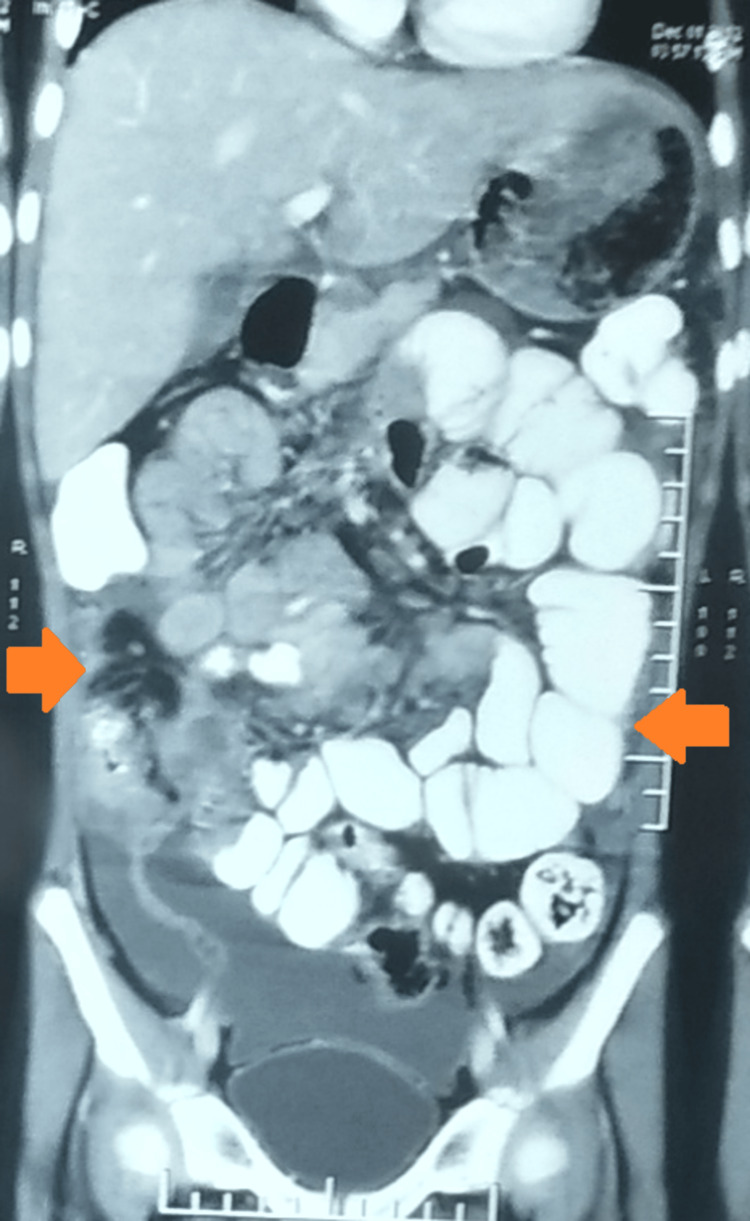
A CECT-whole abdomen showing irregular circumferential thickening involving the terminal ileum, caecum, and part of the proximal ascending colon with multiple discrete enlarged lymph nodes (arrows) CECT: contrast-enhanced computed tomography

An ultrasound of the whole abdomen was unremarkable. As the patient still had abdominal pain, a contrast-enhanced computed tomography of the whole abdomen was done, which revealed irregular circumferential thickening involving the terminal ileum, caecum, and part of the proximal ascending colon, causing narrowing of the lumen with mild dilatation of small bowel loops proximal to it. Multiple discrete enlarged lymph nodes were seen, with a few showing internal necrosis in the periportal, peripancreatic, gastrohepatic, supradiaphragmatic, aortocaval, precaval, and mesenteric regions. Thickening of the peritoneum and omentum with mild ascites was also noted (Figure 3).

**Figure 4:**
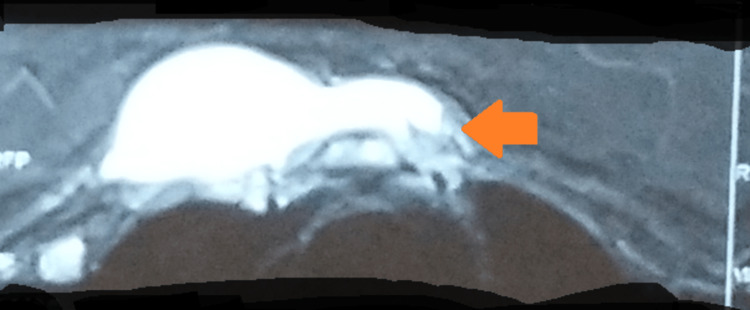
MRI-chest showing a hyperintense, irregular collection anterior to the manubrium sterni (arrow) MRI: magnetic resonance imaging

A magnetic resonance imaging of the chest showed a hyperintense, irregular collection about 100 X 64 X 29 mm anterior to the manubrium sterni and adjoining anterior ribs, largely on the right side. Marrow edema was seen in the underlying manubrium sterni. Mild intercostal extension was seen between the first and second costal cartilages. A subcutaneous tract was seen extending superiorly towards the right supraclavicular region. A mild right-sided pleural effusion was also noted (Figure 4).

A diagnostic pleural tapping was done, and an intercostal drain was inserted, followed by intrapleural fibrinolysis. Results were suggestive of an exudate with lymphocytic predominance. However, Ziehl-Neelsen staining for acid-fast bacilli, Gram staining, and culture were negative, but CBNAAT was positive for *Mycobacterium tuberculosis* with low detection. Furthermore, after five days, this drain was removed and suturing was done. Sutures were removed on the tenth day.

For the right anterior chest wall abscess, ultrasound-guided fine needle aspiration of pus was done using a 16G needle. About 40 cc of pus was drained, and samples were sent for microscopy and CBNAAT. A smear of the pus was indicative of numerous intact and degenerated polymorphs, lymphocytes, plasma cells, and macrophages. No epitheloid granulomas or atypical cells were seen, but the background showed mainly necrotic material. Further, the Ziehl-Neelsen staining for acid-fast bacilli was positive, but the Gram staining was negative. Furthermore, the CBNAAT of the pus was suggestive of low detection of *Mycobacterium tuberculosis* with resistance to rifampicin. Additional samples were sent for culture and drug-susceptibility testing, which grew *Mycobacterium tuberculosis* with resistance to rifampicin. Second-line drug-susceptibility testing was suggestive of fluoroquinolone resistance. The results of the line probe assay and culture of the sputum sample were negative.

Finally, a diagnosis of primary disseminated pre-extensively drug-resistant tuberculosis of the lungs, pleura, chest wall, and abdomen was made, and she was planned for the all-oral longer regimen per the national guidelines and admitted to the drug-resistant tuberculosis center. A detailed pre-treatment evaluation was done (Table 1).

**Table 1:** Pretreatment evaluation according to the national guidelines

Test	Result	Reference range
Hemoglobin	11.1 g/dL	11.9-15
Platelet count	2.2 x 109/L	1.5-4.0 x 109
Total leukocyte count	7.2 × 109/L	4-10
Erythrocyte Sedimentation Rate	60 mm/hr	0-20
Bilirubin (conjugated)	0.2 µmol/L	<1
Human immunodeficiency virus	Non-reactive	Reactive-Non-reactive
Fasting blood sugar	4.10 mmol/L	3.9-5.6
Serum electrolytes (Sodium, Potassium, Magnesium, and Calcium)	Normal	Normal-Abnormal
Serum creatinine	54 µmol/L	53-97.2
Urine routine and microscopic	Normal	Normal-Abnormal
Urine pregnancy test	Negative	Negative-Positive
Serum thyroid-stimulating hormone levels	0.5 mU/L	0.4-4.0
Mental health assessment (Psychiatric issues)	Absent	Present-Absent
Electrocardiogram	Normal	Normal-Abnormal
Ophthalmology opinion	Normal	Normal-Abnormal

Once the pre-treatment evaluation was found to be normal, an all-oral longer regimen was initiated (Table 2). She responded well to the treatment without any major adverse drug reactions and was discharged after two weeks. This patient continued her treatment for a total of 18 months and was finally declared treatment complete. Her last chest radiograph and ultrasound of the abdomen and chest at the eighteenth month were normal.

**Table 2:** An all-oral longer regimen as per her weight

Drug	Dose	Route of administration	Duration	Frequency
Bedaquiline	400 mg	Per oral	2 weeks	Daily
	200 mg	Per oral	22 weeks	Alternate day
Clofazimine	100 mg	Per oral	18 months	Daily
Linezolid	600 mg	Per oral	6 months	Daily
	300 mg	Per oral	12 months	Daily
Levofloxacin	1000 mg	Per oral	18 months	Daily
Cycloserine	750 mg	Per oral	18 months	Daily
Pyridoxine	100 mg	Per oral	18 months	Daily

## Discussion

Tuberculosis is a substantial contributor to morbidity and mortality [4]. The disease is an outcome of infection by *Mycobacterium tuberculosis* [4,6]. Often, pulmonary involvement is seen, but extrapulmonary tuberculosis is also a growing health issue [4,7]. Besides, disseminated tuberculosis is also reported in drug-resistant tuberculosis cases [6].

Pre-extensively drug-resistant tuberculosis looms large in countries like India [8]. It contributes to a significant number of total drug-resistant tuberculosis cases [6,8]. Adwani et al. reported 55.65% of pre-extensively drug-resistant tuberculosis cases among pulmonary multidrug-resistant tuberculosis cases in a tertiary care hospital in Mumbai [9]. Similarly, Singhal et al. reported the extent to be about 49.4% [8].

The diagnosis of primary cases where no previous history is available for this type of bacterial infection is an uphill task [10]. Often, the diagnosis is delayed due to late presentation, improper testing, or a lack of awareness among primary care physicians. A diagnosis of pre-extensively drug-resistant tuberculosis is established by culture and drug susceptibility testing of *Mycobacterium tuberculosis* [8]. This could be done either genotypically, i.e., by first- and second-line line-probe assays, or whole-genome sequencing [8]. Further, phenotypically, it can be achieved by culture-based drug susceptibility testing [10].

Similar to other drug-resistant tuberculosis types, the management of pre-extensively drug-resistant tuberculosis is difficult, mainly due to the social stigma, reluctance of patients to undergo long treatments, adverse drug reactions, and high pill burden [1,6]. The situation becomes very challenging when extrapulmonary involvements, as seen in this case, are present. The World Health Organization recommends a multi-drug regimen for such cases [11]. Still, there is no consensus on the exact treatment duration, especially in extrapulmonary cases [6,11].

Disseminated tuberculosis has a propensity to develop in immunocompromised patients, but reports of immunocompetent cases are also available [12]. The commonest cause of the development of disseminated disease is attributed to the reactivation of an old latent* Mycobacterium tuberculosis* infection [6,12]. Literature suggests that the strongest predictors of mortality are age, delay in presentation, and a grave underlying disease [12].

A detailed literature search revealed that no such case has ever been published where primary disseminated pre-extensively drug-resistant tuberculosis of the lungs, pleura, chest wall, and abdomen is reported. Moreover, the diagnosis of the involvement of these many organs required an extensive diagnostic approach backed by radiometric techniques.

Lastly, a case similar to the present one highlights the importance of a detailed examination and clinical workup. The paucity of data associated with the clinical condition would also help bring out similar reports from endemic areas. This will definitely boost the effort for tuberculosis elimination and help in modifying or making new guidelines.

## Conclusions

Herein, a case of primary disseminated pre-extensively drug-resistant tuberculosis of the lungs, pleura, chest wall, and abdomen is reported. The patient received an all-oral longer treatment for 18 months and was declared treatment complete. The paucity of data related to the topic poses a big challenge for establishing the diagnosis, even in endemic countries. Usually, in high-burden countries, such patients consult primary care physicians, and therefore, it is essential that the latter be trained about such cases to have a high degree of suspicion. This will not only help in timely diagnosis, but it will also prevent unfavorable or fatal outcomes.
